# Nano-Thermal Analysis of Defect-Induced Surface Pre-Melting in 2D Tellurium

**DOI:** 10.3390/nano11102735

**Published:** 2021-10-15

**Authors:** Dae Young Park, Hyang Mi Yu, Byeong Geun Jeong, Sung-Jin An, Sung Hyuk Kim, Mun Seok Jeong

**Affiliations:** 1 Department of Physics, Hanyang University, Seoul 04763, Korea; parkdy004@hanyang.ac.kr (D.Y.P.); ansung5030@skku.edu (S.-J.A.); 2 Department of Energy Science, Sungkyunkwan University, Suwon 16419, Korea; gidal0072@skku.edu (H.M.Y.); zinzza228@skku.edu (B.G.J.); sh.kim@skku.edu (S.H.K.); 3 Department of Energy Engineering, Hanyang University, Seoul 04763, Korea

**Keywords:** 2D Tellurium, nano thermal analysis (Nano TA), surface pre-melting

## Abstract

Thermal properties, such as thermal conductivity, heat capacity, and melting temperature, influence the efficiency and stability of two-dimensional (2D) material applications. However, existing studies on thermal characteristics—except for thermal conductivity—are insufficient for 2D materials. Here, we investigated the melting temperature of 2D Tellurium (2D Te) using the nano-thermal analysis technique and found anomalous behavior that occurs before the melting temperature is reached. The theoretical calculations present surface pre-melting in 2D Te and Raman scattering measurements suggest that defects in 2D Te accelerate surface pre-melting. Understanding the pre-melting surface characteristics of 2D Te will provide valuable information for practical applications.

## 1. Introduction

Two-dimensional (2D) materials, such as transition metal dichalcogenides (TMDCs) and black phosphorene (BP), have received significant interest in both fundamental science and engineering applications owing to their outstanding electronic properties. In particular, they exhibit a direct bandgap and excellent field-effect transistor characteristics, with a high on/off ratio and mobility; hence, they are regarded as promising candidates for various new electronics, optoelectronics, flexible devices, and quantum computing applications [1,2,3,4]. In particular, p-type 2D Tellerium (2D Te)—synthesized via the hydrothermal method—demonstrated excellent electrical properties, such as a fast field-effect mobility of approximately 700 cm^2^·V^−1^·S^−1^ and outstanding thermoelectricity, with a Seebeck coefficient of 413 μV·K^−1^ and a ZT value of 0.63 at room temperature, resulting from high electrical conductivity and low thermal conductivity [5,6,7,8]. 2D Te exhibits several superior properties for many applications, but the thermal properties that can determine the operating temperatures of devices have not yet been elucidated. In addition, the efficiency and stability of 2D Te devices are significantly affected by the thermal properties resulting from the change in the kinetic or potential energies of constituent atoms or molecules of the material [9,10]. Moreover, the degradation of thermal properties from bulk to nanostructure implies that the operating temperature of a device with 2D Te can be more tightly limited. Therefore, it is essential to understand the thermal properties of 2D Te to maximize the performance and lifetime of 2D Te devices.

In this study, we investigated the melting behavior of 2D Te using nano-thermal analysis (Nano TA). Unusual melting characteristics were observed before melting and, through theoretical calculation, were revealed as surface pre-melting. Different surface pre-melting rates were demonstrated in 2D Te for thicknesses of 50 nm and 80 nm. A significant occurrence of surface pre-melting in 50-nm 2D Te was observed. We suggest that the defects on the surface of 2D Te affect the melting properties, resulting in fast surface pre-melting in 2D Te at 50 nm. Raman measurements were conducted to confirm the distribution of defects and the Raman intensity map demonstrated the non-uniformity of the sample surface.

## 2. Materials and Methods

### 2.1. Material and Sample Preparation

Sodium tellurite (Na_2_TeO_3_, 99%) and acetone (ACS reagent, 99.5%) were obtained from Sigma Aldrich (Incheon, Korea). Ammonia solution (35%, extra pure), hydrazine hydrate (80%, wt/wt%), and polyvinylpyrrolidone (PVP, MW 58,000) were purchased from Alfa Aesar (Seoul, Korea). For the 2D Te synthesis, Na_2_TeO_3_ (0.00045 mol, 0.1 g) and PVP (1 g) were dissolved in deionized water (DI), with continuous stirring to form a transparent solution. The solution was transferred to a 100-mL Teflon lined stainless steel autoclave and ammonia solution and hydrazine hydrate were added at a 2:1 volume ratio. The autoclave was tightly sealed and placed in a reaction oven at 180 °C for 40 h. After the reaction, the transparent solution changed to a silver-grey color. For the thinning process (chemical exfoliation), acetone and crude colloid were mixed in a 3:1 volume ratio and allowed to stand for 4 h. After the reaction, exfoliated 2D Te was centrifuged at 5000× *g* for 5 min. The supernatant was discarded and the precipitate was redispersed in DI water. This process was repeated several times to remove unreacted chemicals. The 2D Te colloid was diluted to obtain a proper sample density.

### 2.2. Atomic Force Microscopy (AFM) and Nano-Thermal Analysis (Nano TA)

The samples were scanned in contact mode at a scan rate of 0.1 Hz using a gold-coated atomic force microscopy (AFM) tip (Anasys Instruments, Santa Barbara, CA, USA). Thermal images and AFM images were simultaneously recorded for the same regions. The heating temperature range was 50–400 °C.

### 2.3. Characterization

All measurements were performed at room temperature (21 °C). Confocal Raman scattering (Nanobase, Seoul, Korea) was used to excite the sample with a continuous-wave laser (λ = 532 nm), which was focused using a 40× objective lens (NA = 0.75). X-ray diffraction (XRD) was performed using a Smartlab instrument (Rigaku, Tokyo, Japan) with Cu Kα radiation (0.14506 nm).

## 3. Results and Discussion

**Figure 1:**
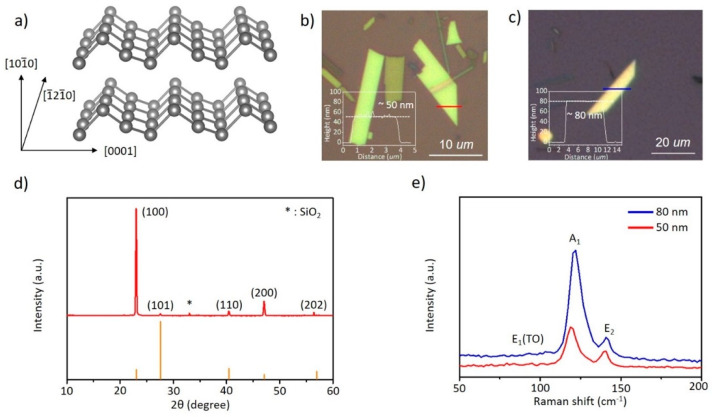
2D Tellurium by hydrothermal method. (**a**) Crystal structure of 2D Tellurium; (**b**,**c**) Optical images and AFM results of 2D Tellurium with 50-nm and 80-nm thickness, respectively; (**d**) X-ray diffraction with simulation; (**e**) Raman scattering according to (**b**,**c**).

2D Te was synthesized via a previously reported hydrothermal method and its structure was characterized prior to nano-thermal analysis (Nano TA) [5]. According to previous computational simulations, Te exhibits three different 2D structures, i.e., *α, β,* and *γ,* which form a spiral atomic chain along the [0001] direction [11,12]. The *α* and *γ* structures are equal to the 1T (octahedral) and 2H (trigonal prismatic) structures of transition metal dichalcogenides, respectively. Meanwhile, *β* has the same crystal structure of black phosphorus (BP), shown in Figure 1a [13,14]. Hydrothermally synthesized 2D Te is formed in the *β* structure. Furthermore, the anisotropy of the crystal structure induces anisotropic electrical transfer and anomalous vibrational behavior [5,15,16,17]. After the thinning process, 2D Te was drop-cast on a SiO_2_ (300 nm)/Si substrate. The optical images in Figure 1b show the trapezoid-shaped 2D Te, in addition to showing wires and dots of Tellurium. At the initial synthesis step, Te was formed as nanowires along the [0001] direction and, with sufficient reaction time, was further grown into the 2D structure. Owing to the growth mechanism, the spontaneous formation of 1D and 2D mixtures is inevitable. Additionally, Te particles were formed by the kinetic energy of OH^−^ ions during the thinning process of 2D Te [18,19]. Figure 1d shows the X-ray diffraction (XRD) results of 2D Te with simulation data. The strong diffraction peaks of the (h00) family planes were mainly observed, indicating a 2D structure [20]. According to previous report [21], Trigonal structure of 2D Te is stable under 5 Gpa and phase transition was not observed before melting. Thus, the thermal property of 2D Te can be evaluated without phase transition effect. In the Raman scattering shown in Figure 1e, three active modes—one A mode and two E modes—were observed at approximately 120.34 cm^−1^ (A_1_), 93.50 cm^−1^ (E_1_ transverse (TO) phonon), and 139.23 cm^−1^ (E_2_), respectively [5,17]. The A_1_, E_1_(TO), and E_2_ modes correspond to chain expansion in the basal plane, bond-bending around the (110) direction, and asymmetric stretching along the helical Te chain, respectively [17,22,23]. The Raman modes of TMDCs change as a function of thickness; similarly, the Raman modes of 2D Te also change depending on thickness; therefore, our Raman scattering results are consistent with previous reports [5,17,22,23].

**Figure 2:**
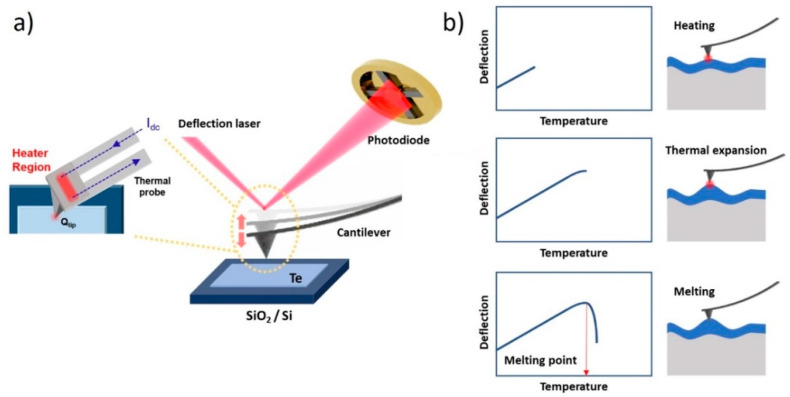
(**a**) Schematic and (**b**) principles of Nano TA.

Nano TA measurements were conducted to analyze the thermal properties of the 2D Te. A schematic and the principle of the Nano TA are shown in Figure 2. The Nano TA probe is used to generate AFM images and allows the user to identify points of local thermal characteristics. The heating temperature in the TA tip in accordance with the applied current increased linearly over time and heat was transferred to the sample. The thermal expansion of the material at the end of the tip was monitored by the change in the incident position of the deflection laser from the oscillation of the tip. At the phase transition temperature (melting or softening, depending on the material), the material under the tip softens and the probe penetrates the sample. This process provides the nanoscale equivalent of a bulk thermo-mechanical analysis experiment, measuring the phase transition temperatures of the sample [24,25,26,27]. The calibration of the heating voltage and tip temperature was performed with a reference sample; the results are presented in Figure S1. To minimize the interaction and incorporation effect of each measurement, the measurement positions of Nano TA have been conducted with a few micrometers’ intervals.

**Figure 3:**
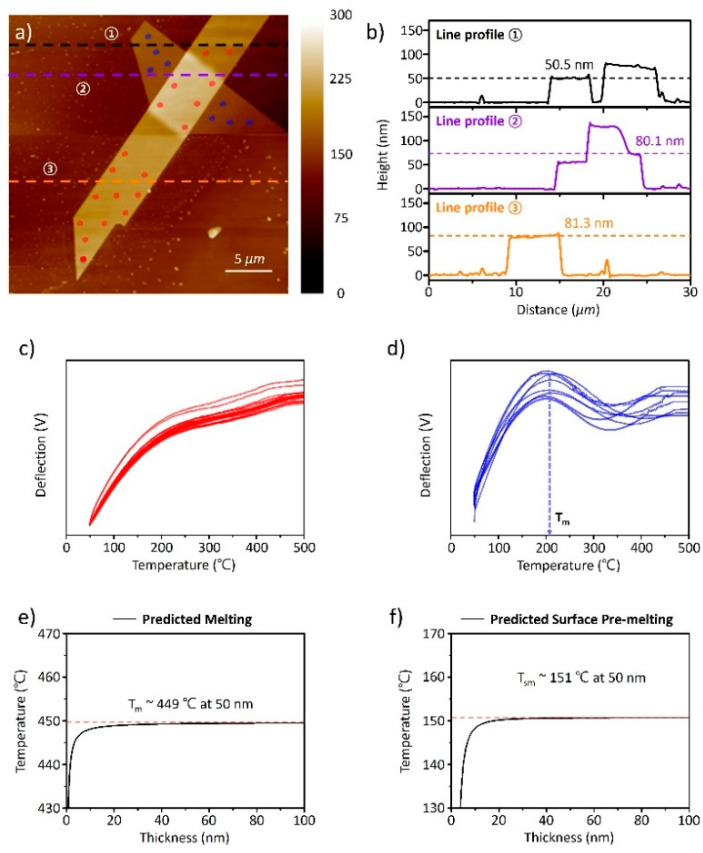
AFM combined Nano TA of 2D Tellurium. (**a**) AFM images and (**b**) three different line profiles are shown in (**a**); (**c**,**d**) Nano TA curves of 2D Tellurium according to different thicknesses presented in (**a**); Models for predicting (**e**) melting point and (**f**) surface pre-melting point for 2D Tellurium.

Figure 3 presents the experimental and theoretical results of Nano TA and modeling for 2D Te with two different thicknesses. According to the line profiles of the AFM image at three other points shown in Figure 3a, the thickness of 2D Te stacked in the vertical direction is measured as 50 nm and 80 nm (Figure 3b). Each curve shown in Figure 3c (red lines) and d (blue lines) indicates the results depending on positions presenting in Figure 3a. In Figure 3c,d, the thermal behaviors of the nonlinear deflection curve, melting, and volume expansion were observed. The constant value of deflection above 400 °C is the out-of-range signal because the measurement ranges of Nano TA are up to 400 °C.

Generally, a proportional relationship between heating temperature and deflection is observed before the phase transition, as shown in Figure 2b. Nonlinear deflection curves usually result from the complex thermal properties of mixed materials [28,29]. For the preparation of 2D Te via the hydrothermal method, polyvinylpyrrolidone (PVP) was added as a capping ligand for the 2D structure of Te [5]. However, the amount of capping ligand in 2D Te is small, resulting in mixed thermal behavior. In addition, the apparent deflection changes in 2D Te are more evident in the 50 nm sample than in the 80 nm sample, although 2D Te with 50 nm thickness contains less PVP content than the 80 nm sample. This indicates that the unique features of deflection are entirely attributed to the 2D Te. Interestingly, both samples exhibited a change in the inclination of the deflection curves near 150 °C. However, a decline in the deflection curves in 50-nm-thick 2D Te was clearly observed, indicating the melting of materials, whereas ambiguous deflection curves in 80 nm thick 2D Te were exhibited. To clarify the temperature for melting 2D Te with a thickness of 50 nm, we conducted the differential function of the deflection curves; the results are shown in Figure S2 and the exact values are given in Table S1. The melting temperature of 50 nm thick 2D Te is approximately 210 °C, which is significantly lower than that of bulk Te (449.5 °C) [30]. Therefore, we estimated that the melting of 2D Te began at approximately 150 °C, but the difference in melting rate appeared as a gradient change in inclination (nonlinearity) and the decline of deflection curves in the 80 nm and 50 nm samples, respectively. Afterward, the deflection curves increased again after 350 °C, which can be understood by the thermal expansion of the materials after the phase transition.

Through the Nano TA measurement, the melting of 2D Te at different rates was observed below the melting temperature of the bulk Te. The thermal properties, including the melting point, deteriorated as the size of the nanomaterial decreased [31]. To understand the unique features of 2D Te, we adapted the model for changing melting points with variations in size and shape. In a previous report, the melting point model of a nanomaterial was demonstrated for three representative shapes: nanoparticles (0D), nanowires (1D), and nanofilms (2D) [32]. We assumed 2D Te to be a nanofilm with a trigonal crystal structure and calculated the model. The detailed process is described in Supporting Note 1. The calculation results for the melting point are shown in Figure 3e. Interestingly, the calculated melting point of 2D Te dramatically drops at thicknesses below 10 nm, whereas the melting point does not change significantly at thicknesses exceeding 10 nm.

The inconsistency in the model for melting point and the experimental feature indicates that the melting of 2D Te below the bulk melting temperature does not originate from the deterioration of the thermal properties of nanomaterials [32]. However, the distinctive melting properties at low temperatures can be explained by surface pre-melting. Surface pre-melting, the initial melting step, formed a thin liquid layer at the surface below the melting temperature (*T_m_*). This feature is frequently observed in both bulk and nano-materials [33,34,35,36]. As various models for melting points have been developed, surface pre-melting models have been studied [37]. We applied the surface pre-melting model for our case and the detailed process is presented in Supporting note 2 [38]. In Figure 3f, the surface pre-melting is presented as a function of varying 2D Te thickness. The calculated *T*_sm_ is approximately 423 K (~150 °C), which is below the observed *T_m_* of 210 °C. This is in good agreement with the temperature of the inclination change in the deflection of 2D Te.

Factors such as dimensionality, size, surface defects, and crystallinity determine the surface pre-melting rate of materials [35,39]. Among these, defects—such as grain boundaries, dislocations, and vacancies—significantly influence the surface pre-melting because the free energy of the solid–solid interface is more significant than that of the two solid–liquid interfaces, leading to pre-melting near the defects [35,36]. Thus, the different rates of surface pre-melting in 2D Te can be explained by the defect density on the surface due to the enormous influence of surface defects. To obtain thin 2D Te, 2D Te was exfoliated by hydroxy ions (OH^−^), similar to the chemical exfoliation of TMDCs with butyllithium treatment, causing damage to the kinetic energy of the mobile ions [18,19]. The many dots observed in the AFM image of Figure 3a result from the favorable formation of dots by the highly oriented chain of Te [5,14,17]. In addition, defects such as Te vacancies and grain boundaries on the surface were spontaneously generated by the formation of Te fragments [18,19]. Therefore, we hypothesize that the surface defect densities on the two samples are different.

**Figure 4:**
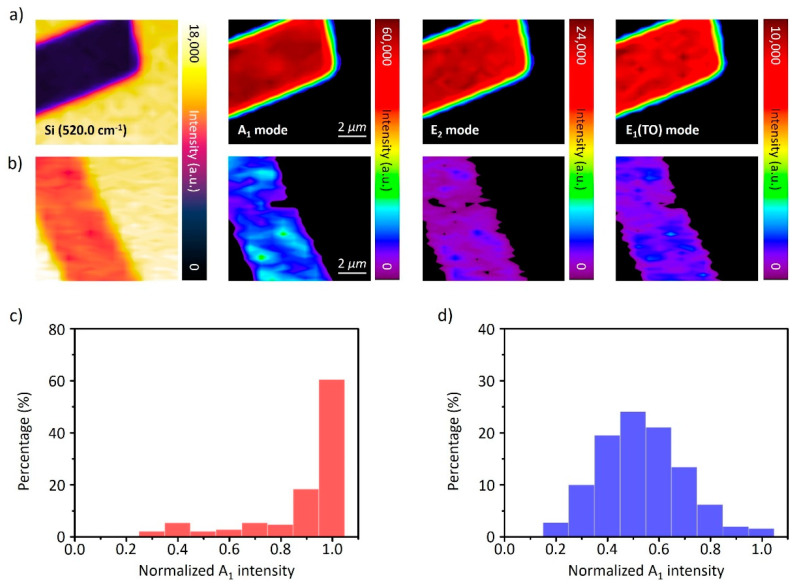
Raman intensity mapping images of A_1_, E_2_, and E_1_ (TO) at the different thicknesses of 2D Tellurium. (**a**) 80 nm and (**b**) 50 nm; (**c**) Histogram of normalized A_1_ mode intensity according to (**a**); (**d**) Histogram of normalized A_1_ mode intensity according to (**b**).

Raman mapping was conducted to evaluate the defects in the 2D Te. Raman scattering is widely used to evaluate material quality, concentration, doping, thickness, and defects [40,41,42,43]. Particularly, as Raman intensities are proportional to material thickness, the surface roughness can be estimated using the difference in Raman intensity [42]. Furthermore, the wide distribution of Raman intensity directly correlates with the density of surface defects. Raman mapping was conducted using a 532-nm excitation laser with a 0.1 s exposure time to check the formation of surface defects in 2D Te after the thinning process. The maximum intensities of the Si Raman mode are shown in Figure 4a,b, and these can be directly compared with each other. The strong Si Raman intensity in the Te area indicates that Te is thin with a rough surface due to exfoliation. The intensity mappings of the A_1_ vibrational mode in 2D Te with different thicknesses of 50 nm and 80 nm are shown in Figure 4a,b, respectively. The A_1_ mode of Raman scattering in 80 nm is strong and uniform, whereas in 50-nm 2D Te, that is uneven. Similarly, full width at half maximum (FWHM) value of A_1_ Raman mode at 80-nm 2D Te shown in supporting Figure S3 demonstrated narrower than 20-nm 2D Te. The uniformity of Raman intensity and narrow FWHM value of Raman mode indicate the less exfoliated 2D Te has a smooth surface, with uniform thickness and high crystallinity, because the area damaged by hydroxyl ions is small [18,19,44]. Accordingly, the strong intensity, uniformity and narrow FHWM of the A_1_ vibrational mode present a slow rate of surface pre-melting. The other Raman modes of 2D Te (E2 and E1(TO)) also show similar trends with respect to thickness, as shown in Figure 4a,b. To precisely analyze the differential of defect density in 2D Te with 50 nm and 80 nm thicknesses, a comparison of the relative A_1_ Raman intensity was conducted, and the histograms of the relative A_1_ intensity are shown in Figure 4c,d. The percentages of the more populated ranges in the histograms are 81.82% from 0.8 to 1.0 (80 nm) and 70.23% from 0.4 to 0.6 (50 nm). The standard deviations of these ranges are 24.34 and 27.07. The higher standard deviation for 50 nm 2D Te indicates a higher surface defect density than that of 80-nm 2D Te. This accelerated the surface pre-melting in the 50-nm Te, leading to a decline in deflection. Afterward, the thermal expansion of molten Te on the surface induces an increased deflection [35]. However, there is no observation of pre-melting phenomena at 80-nm 2D Te owing to the slow surface pre-melting rate [39].

## 4. Conclusions

In conclusion, the unnatural melting behavior of 2D Te below *T_m_* was observed through Nano TA measurements. Theoretical modeling was conducted to determine the origin of the phenomena, which was found to be surface pre-melting, and the hypothetical value was derived at approximately 150 °C, similar to the experimental results. Surface pre-melting was slowly observed in 2D Te at 80 nm, whereas it drastically occurred in the 50-nm sample. The different rates of surface pre-melting in 2D Te were estimated as the density of surface defects formed by solvent exfoliation. This was confirmed by the intensity variation of Raman scattering, which is an indicator of the surface defect density. We found that the surface pre-melting of 2D Te at 150 °C can lead to unpredictable destruction of the Te device resulting from defects formation on the surface. The observation of surface pre-melting will provide a better understanding of the fundamental physical properties of other 2D mono-element materials including 2D Te which has received intensive interest as a fascinating material in electronics and thermoelectric fields.

## Data Availability

Data presented in this study are available on request from the corresponding author.

## Supplementary Materials

The following are available online at https://www.mdpi.com/article/10.3390/nano11102735/s1, Figure S1: Nano TA calibration curves, Figure S2: Derivative curves of Nano TA plot in Figure 3d, Figure S3: FWHM of A_1_ Raman mode in 2D Te with different thickness. (a) 80 nm and (b) 20 nm, Table S1: Values of fitted functions in Figure S2, Supporting note 1: Calculation of the melting of 2D Te depending on the thickness, Supporting note 2: The model for surface pre-melting of 2D Te depending on thickness.
