# Supplementary material for: Nano-Thermal Analysis of Defect-Induced Surface Pre-Melting in 2D Tellurium
Source: Nanomaterials (Basel). 2021 Oct 15;11(10):2735. doi: 10.3390/nano11102735 (PMC8541556; doi:10.3390/nano11102735)
Supplement: Supplementary file 1 [file nanomaterials-11-02735-s001.zip › nanomaterials-1373957-supplementary.pdf]

nanomaterials

Supplementary Materials

Nano-Thermal Analysis of Defect-Induced Surface Pre-Melting in 2D Tellurium

Dae Young Park [1,†], Hyang Mi Yu [2,†], Byeong Geun Jeong [2,†], Sung-Jin An [1], Sung Hyuk Kim [2] and Mun Seok Jeong [1,3,*]

1 Department of Physics, Hanyang University, Seoul 04763, Korea; parkdy004@hanyang.ac.kr (D.Y.P.); ansung5030@skku.edu (S.-J.A.)
2 Department of Energy Science, Sungkyunkwan University, Suwon 16419, Korea; gidal0072@skku.edu (H.M.Y.); zinzza228@skku.edu (B.G.J.); sh.kim@skku.edu (S.H.K.)
3 Department of Energy Engineering, Hanyang University, Seoul 04763, Korea
* Correspondence: mjeong@hanyang.ac.kr
† These authors contributed equally to this work.

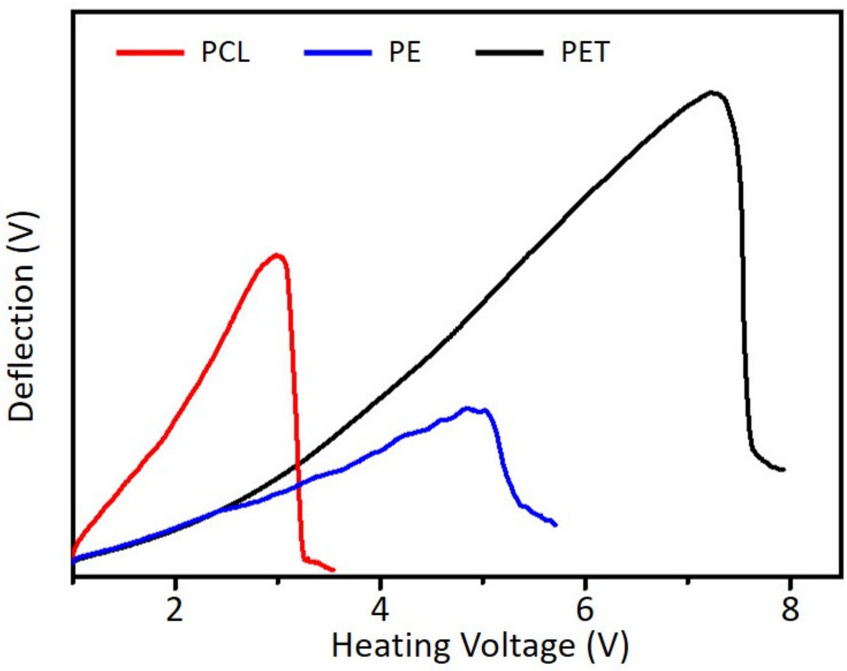

Figure S1. Nano TA calibration curves.

The reference samples are polycaprolactone (PCL), polyethylene (PE), and polyethylene terephthalate (PET), obtained from Anasys Instruments (Santa Barbara, CA, USA). The bending points of the heating voltage are the melting points of the reference samples.

Note that the Nano TA is a powerful tool that combines atomic force microscopy (AFM) and photo-thermal induced resonance spectroscopy (PTIR) to probe and correlate aspects of the film topology and thermal absorption mapping images of the local region at a specific frequency. However, we cannot evaluate the effect of further process such as annealing for removal of defects because of the sample destruction after measurement and focused on intrinsic property.

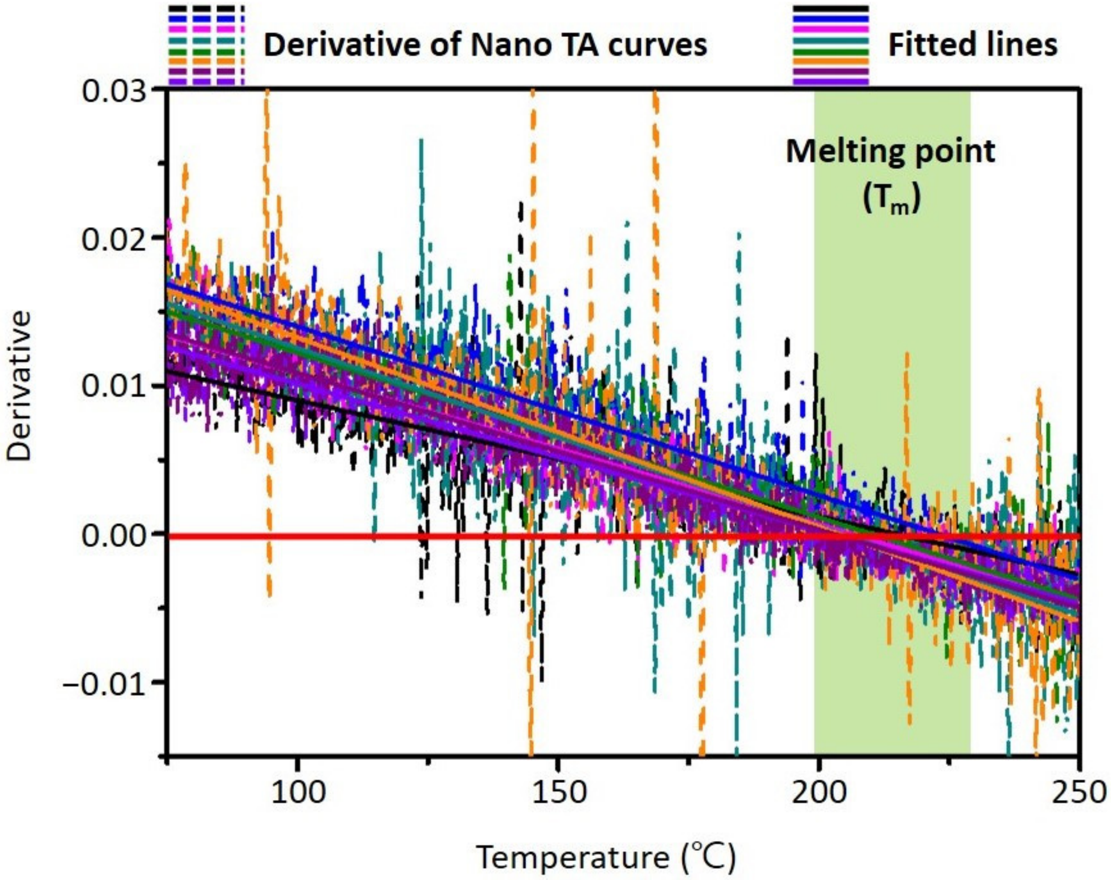

Figure S2. Derivative curves of Nano TA plot in Figure 3d.

To determine the exact meting point of 2D tellurium (Te), Nano TA curves were differentiated to the first order. There is little difference among the eight measurement points and the variation in melting point is not significant.

Table S1. Values of fitted functions in Figure S2.

Position	Function y = a + bx	Values	X intercept (Tm, °C)
1	a	0.01687	214.92
	b	−0.00008	
2	a	0.02540	222.94
	b	−0.00011	
3	a	0.02108	206.15
	b	−0.00010	
4	a	0.02333	210.11
	b	−0.00011	
5	a	0.02457	204.43
	b	−0.00012	
6	a	0.02603	203.78
	b	−0.00013	
7	a	0.02002	202.52
	b	−0.00010	
8	a	0.02145	202.88
	b	−0.00011	

Supporting note 1. Calculation of the melting point of 2D Te depending on the thickness.

The general equation is shown below:

$$\frac{T_{mn}}{T_{mb}} = 1 - 2(1-q)\left(\frac{3-\lambda}{3}\right)\frac{aX}{b+\left(\frac{3-\lambda}{3}\right)aX},\ a = 2P_S d,\ b = P_L,\ X = \frac{1}{size},$$

where T_{mn} and T_{mb} are the melting points of the nanomaterials and bulk, respectively, λ is the shape of the nanomaterial (λ = 0 for the particle, λ = 1 for the nanowire, and λ = 2 for the nanofilm), and X is the reciprocal of the nanomaterial size (diameter of the nanoparticle and nanowire, and film thickness for the nanofilm). q is the surface-to-volume coordination number ratio, where $q = \beta_S/\beta_L$ (S: surface, L: lattice). P_S and P_L are the packing fractions of the surface crystalline plane and lattice, respectively.

The 2D Te has a trigonal crystal structure (space group No. 152, $P3_121$) with a = b = 4.514 Å, c = 5.955 Å, and α = β = 90 ° and γ = 120 °. The parameters for the model were calculated using the following process.

The cell volume of trigonal (104.72 Å^3) can be calculated using the equation $V = a^2 c\sin(60^o)$. The packing fraction of the lattice (P_L) is the ratio of the occupied atoms to the cell volume. The diameter of the Te atom (d) is 1.4 Å (V = 11.49 Å^3) and P_L was calculated as 0.328. The surface crystalline plane of Te is (100), and the packing fraction on the surface crystalline plane (Ps) was calculated as 0.229. The coordination numbers of the lattice (β_L) and surface crystalline plane (β_S) are 12 and 2, respectively. Therefore, the combined parameters a ($2P_S d$) and q (β_L/β_S) are 1.283 Å and 0.1667, respectively. The assumption is that 2D Te is a nanofilm, λ = 2. The melting point of bulk Te is 722.66 K.

Supporting note 2. The model for surface pre-melting of 2D Te depending on thickness.

We adopted the surface pre-melting model presented by Zhou et al. The equation is as follows:

$$T_{sm}(d) = \left(\frac{3}{4}a\right)^{-1}\{1+y(d)\}e^{-y(d)}T_m(\infty),\ a = \frac{2S_{vib}(\infty)}{3R}+1,\ y(d) = \frac{(a-1)}{(\frac{d}{2r_0}-1)},$$

where T_{sm} and $T_m(\infty)$ are the temperatures of the surface pre-melting and bulk melting, respectively. S_{vib} is the vibrational component of melting entropy defined as $S_{vib} = S_m - R$, where S_m and R (8.314463 J·mol^{-1}K^{-1}) are the melting entropy and ideal gas constant, respectively. r_0 is the nanoparticle radius when all atoms are located on the surface. d is the size of the nanoparticles. To consider the shape as similar to the model for the melting point of 2D Te, we slightly modified the size term $\frac{d}{2r_0}$ to $\frac{t}{2d}$, where t and d (0.4 nm) are Te films and monolayers, respectively.

The required parameter for the prediction of the surface pre-melting temperature in 2D Te is the melting entropy (S_m), defined as $S_m = \frac{\Delta H_{fus}}{T_f}$, where H and T_f are the enthalpy and temperature of fusion (17.49 kJ·mol^{-1} and 722.66 K). Using this parameter, we calculated the surface pre-melting temperature of the 2D tellurium as a function of thickness.

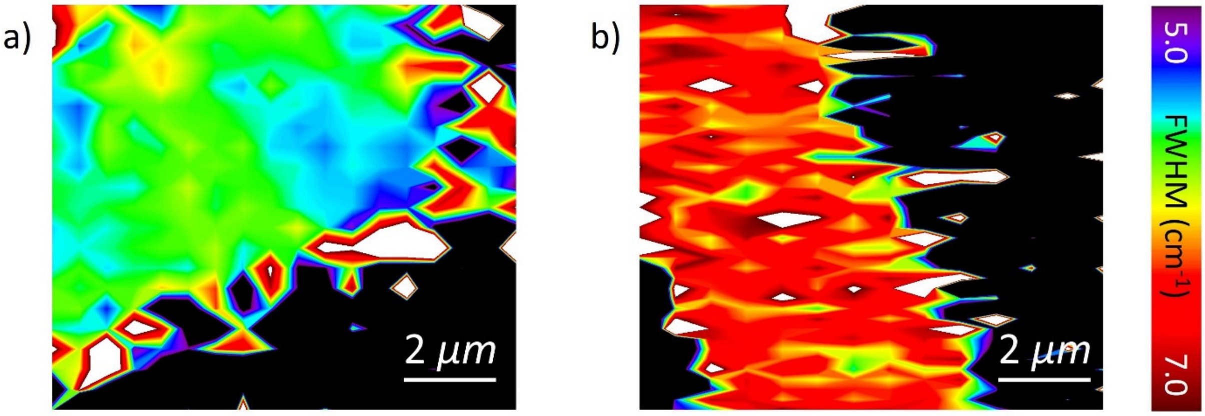

Figure S3. FWHM of A_1 Raman mode in 2D Te with different thickness. (**a**) 80 nm and (**b**) 20 nm.
